# Maternal exposure to PM_2.5_ may increase the risk of congenital hypothyroidism in the offspring: a national database based study in China

**DOI:** 10.1186/s12889-019-7790-1

**Published:** 2019-11-19

**Authors:** Li Shang, Liyan Huang, Wenfang Yang, Cuifang Qi, Liren Yang, Juan Xin, Shanshan Wang, Danyang Li, Baozhu Wang, Lingxia Zeng, Mei Chun Chung

**Affiliations:** 1 Department of Obstetrics and Gynecology, Maternal & Child Health Center, the First Affiliated Hospital of Xi’an Jiaotong University, No. 277, Yanta West Road. Xi’an, Shaanxi Province 710061 Xian, People’s Republic of China; 2 School of Public Health, Xi’an Jiaotong University Health Science Center, Xi’an, Shaanxi People’s Republic of China; 3 Department of Public Health and Community Medicine, Tufts University School of Medicine, Massachusetts Boston, USA; 4 Department of Women’s and Children’s Health, Karolinska Institute, Solna, Stockholm, Sweden; 5 Northwest Women’s and Children’s Hospital, Xi’an, Shaanxi People’s Republic of China

**Keywords:** Particulate matter, Air quality index, Congenital hypothyroidism, Cut-off value

## Abstract

**Background:**

Maternal exposure to air pollution is related to fetal dysplasia. However, the association between maternal exposure to air pollution and the risk of congenital hypothyroidism (CH) in the offspring is largely unknown.

**Methods:**

We conducted a national database based study in China to explore the association between these two parameters. The incidence of CH was collected from October 1, 2014 to October 1, 2015 from the Chinese Maternal and Child Health Surveillance Network. Considering that total period of pregnancy and consequently the total period of particle exposure is approximately 10 months, average exposure levels of PM_2.5_, PM_10_ and Air Quality Index (AQI) were collected from January 1, 2014 to January 1, 2015. Generalized additive model was used to evaluate the association between air pollution and the incidence of CH, and constructing receiver operating characteristic (ROC) curve was used to calculate the cut-off value.

**Results:**

The overall incidence of CH was 4.31 per 10,000 screened newborns in China from October 1, 2014 to October 1, 2015. For every increase of 1 μg/m^3^ in the PM_2.5_ exposure during gestation could increase the risk of CH (adjusted OR = 1.016 per 1 μg/m^3^ change, 95% CI, 1.001–1.031). But no significant associations were found with regard to PM_10_ (adjusted OR = 1.009, 95% CI, 0.996–1.018) or AQI (adjusted OR = 1.012, 95% CI,0.998–1.026) and the risk of CH in the offspring. The cut-off value of prenatal PM_2.5_ exposure for predicting the risk of CH in the offspring was 61.165 μg/m^3^.

**Conclusions:**

The present study suggested that maternal exposure to PM_2.5_ may exhibit a positive association with increased risk of CH in the offspring. We also proposed a cut-off value of PM_2.5_ exposure that might determine reduction in the risk of CH in the offspring in highly polluted areas.

## Background

Rapid economic development and increasing urbanization are mainly responsible for increased air pollution emissions. Air pollution has become a global environmental burden since currently 92% of the world’s population lives in high-polluted areas where air pollution level usually exceeds the stated values of WHO guideline [1]. As a result of rapid industrialization that occurred in the past two decades, China is heavily affected by air pollution. It was reported that the annual mean concentration (standard deviation [SD]) of PM_2.5_ was 51.5 (21.6) μg/m^3^ in China from 2014 to 2016, which is considerably higher than the standard guidelines (10 μg/m^3^ for PM_2.5_) of the WHO [2, 3]. In addition, the exposure levels of other pollutants have been reported to exceed the levels specified by the WHO guideline [4, 5].

In recent years, the adverse effects of environmental pollution on public health have been studied extensively. Several studies have reported that air pollutants can enter the human body through the respiratory tract and may cause inflammation reaction and oxidative stress, etc. [6], which may increase the risk for the development of cardiovascular and respiratory diseases [7–9]. This has led to more than 3 million premature deaths globally each year [10–14]. As a result of the increase in the tidal volume and respiratory rate [15], pregnant women are at higher risk from exposure to air pollution than non-pregnant women. Furthermore, embryo and fetal development are highly susceptible to air pollutants, notably particulate matter (PM), which can enter the placenta through the respiratory tract and the maternal blood. PM_2.5_ is more likely to contain harmful substances, such as polycyclic aromatic hydrocarbons (PAHs), heavy metals [16–18]. These substances might pose further threat to maternal and fetal health including gestational diabetes, birth defects and low birth weight [19–23]. Among these disorders, the impact of PM on fetal development has received considerable attention.

Previous studies regarding the effects of air pollution on fetal development have focused on fetal growth and birth defects. However, the effects of air pollution on fetal thyroid function have been investigated to a lesser extent. The development of fetal thyroid function is crucial for the fetus and congenital hypothyroidism (CH) can be caused if this function is inhibited. And autoimmune conditions such as hypothyroidism, diabetes and immune mediated allergic reaction often present with subtle signs and therefore requires a high index of suspicion for diagnosis and prevention strategies [24, 25]. CH exhibits a worldwide annual incidence of 1 in 2000–4000 live births [26]. In China, its incidence varies across provinces. According to nationwide statistics, the incidence of CH in Xinjiang (a province located in the northwest of China) in 2015 was 1 in 5327 live births, while Zhejiang (a province located in the southeast of China) reached an incidence rate of 1 to 1347 live births [27]. Several studies indicated that CH was considered as one of the most common preventable causes of mental retardation [28]. Certain irreversible complications for infants, such as neurological damage, slow growth, delayed skeletal maturation and mental retardation might be caused in case of untimely treatment of CH [29–32]. Therefore, the exploration of additional CH risk factors, such as air pollution plays a crucial role in reducing the incidence of CH and improving the quality of life in the offspring. However, to date only one study has explored the correlation between maternal air pollution exposure during gestation and fetal thyroid function. It was suggested that PM_2.5_ exposure during the third trimester of gestation was associated with lower TSH levels and the FT4/FT3 ratio in cord blood. Consequently, the effects of air pollution exposure during gestation on the thyroid function of fetuses and infants require further investigation, notably in highly polluted areas.

Since 2013, China has been gradually including PM_2.5_, PM_10_ and other air pollutants in the national air quality monitoring network and in the public real-time monitoring data. In addition, the annual incidence of CH was recently summarized and published by the National Office of Maternal and Child Health Surveillance. Therefore, a national study was conducted to examine the association between annual exposure levels of PM_2.5_, PM_10_ and air quality index (AQI) in 2014 with the annual incidence of CH from October 1, 2014 to October 1, 2015.

## Methods

### Study design

A database based study was conducted to analyze the association between maternal exposure to air pollution and the incidence of CH in 30 provinces of China. Average incidence of CH in 30 provinces were collected from the October 1, 2014 to October 1, 2015. Considering that the exposed window of pregnancy is approximately 10 months, average exposure levels of PM_2.5_, PM_10_ and AQI in 30 provinces were collected from January 1, 2014 to January 1, 2015, and then calculated the relationship between air pollution exposure and the risk of CH in the offspring.

### Data resource

#### CH newborn screening

The annual incidence of CH in 30 provinces of China from October 1, 2014 to October 1, 2015 was collected in the present study. The data was derived from the annals on the Chinese neonatal metabolic disease screening in 2015, which was compiled by the National Office of the Maternal and Child Health Surveillance (NOMCHS) based on the Chinese Newborn Screening information System (CNSIS), and published in the Chinese Maternal and Child Health Surveillance Network in February 2017 [27]. CNSIS is a screening program for newborns performed across the country, including the screening of CH. Whole-blood was collected from every newborn on filter papers for measuring the serum levels of thyrotropin (TSH) between 72 h and 7 days following birth. While premature, low birth weight or sick neonates and those who were discharged early within 7 days of birth were sampled within 20 days after birth. Any cases which indicated elevated TSH levels (higher than 10 to 20 μIU/ml) through double testing were followed-up and subjected to further diagnosis. Finally, the diagnosis of CH was confirmed by trained pediatric endocrinologists based on serum thyroid function tests [increased serum TSH, and reduced serum free thyroxine (FT4)]. And each newborn diagnosed with CH was reported to local newborn screening centers (LNBSCs). Through the summary, annual screening results of each province were published on the public website by NOMCHS finally. The screening and diagnosis of the newborns adhered to the Technological Guideline on National Newborn Screening (2010) issued by the Ministry of health of the people’s republic of China. In addition, a written informed consent on CH screening was obtained from the neonates’ parents prior to the collection of their blood samples.

#### Exposure assignment

Considering that the gestation period is approximately 10 months, we collected and summarized the monthly average levels of PM_2.5_, PM_10_ and AQI in cities of 30 provinces (with the exception of Tibet, Taiwan, Hong Kong and Macau) of China from January 1, 2014 to January 1, 2015. In the present study, ambient air pollution exposure data were provided by the Chinese Air Quality Online Monitoring and Analysis Platform [33], which had collected and released real-time and historical data on PM_2.5_, PM_10_ and AQI exposure levels. The data of this website originated mainly from the real-time data of ambient air monitoring which was recorded by the Ministry of Ecology and Environment of the People’s Republic of China [34]. Each pollutant was continuously measured, PM_2.5_ was measured by β ray and Tapered Element Oscillating Microbalance, and PM_10_ was measured by light scattering methods.

### Covariate factor

Taking into account the difference of economy and other pollution factors among 30 provinces, our study also considered including per capital gross regional product (GRP) and the content of heavy metals [including lead (Pb), mercury(Hg), arsenic (As)] in wastewater in 30 provinces from January 1, 2014 to January 1, 2015 as potential covariates factors. Those indicators were collected from China Statistical Yearbook [35, 36] and incorporated into models as potential confounding factors.

### Statistical analysis

GeoDa version 1.10 was used to visualize the average incidence of CH in 30 provinces from October 1, 2014 to October 1, 2015. The average exposure values of PM_2.5_, PM_10_ and AQI from January 1, 2014 to January 1, 2015 in each province were estimated based on monthly average exposure records in all cities of each provinces by the weighted average method. Spearman’s rank correlation analysis based on non-parameter was used to analyze the correlations among each indicators. Generalized additive model (GAM) was used to estimate the nonlinear relationship between air pollution exposure during pregnancy and the incidence of CH. Poisson distribution was fitted in GAM model because the incidence of CH was qualitative data and belonged to small probability event. The basis GAM equation is listed as below,

$$ \log \left[E\left({Y}_t\right)\right]=\alpha +{\sum}_{i=1}^n{\beta}_i{X}_i+{\sum}_{j=1}^m{f}_j\left({Z}_j\right) $$

In this equation, *E*(*Y*_*t*_) is the expected incidence of CH in province t, *α* is the intercept and β is the parameter vector that predict the effect of air pollutants (PM_2.5_, PM_10_, AQI); *X*_*i*_ represents the exposure concentration of air pollutants (PM_2.5_, PM_10_, AQI) in province t; *f*_*j*_(*Z*_*j*_) is natural spline function, and *Z*_*i*_ represents the exposure value of covariate factors in province t, including per capital GRP, the content of heavy metal in wastewater(Pb, As, Hg); df means the degrees of freedom. Based on Akaike information criterion (AIC), the degrees of freedom of covariate were determined. Then, the single pollutant model was built as follow:

$$ \mathrm{Log}E\left({Y}_t\right)=\beta {X}_t+\mathrm{ns}\left(\mathrm{PCGRP}, df=7\right)+\mathrm{ns}\left(\mathrm{Pb}, df=3\right)+\mathrm{ns}\left(\mathrm{As}, df=3\right)+\mathrm{ns}\left(\mathrm{Hg}, df=5\right)+\alpha $$

In the basic model, single pollutant was gradually added to evaluate the impact of each 1 unit increase of air pollution exposure on the incidence of CH.

Furthermore, 30 provinces was divided into high-incidence areas and low-incidence areas according to the 50th quantile of CH incidence (3.615 per 10,000 live births). Independent sample T test was used to compare the differences of pollutant indicators between those two areas. And then the cutoff value of air pollution for predicting CH and its sensitivity and specificity were calculated by constructing receiver operating characteristic (ROC) curves and Youden index which is equal to the sum of sensitivity and specificity minus 1. In addition, we grouped the 30 provinces into high-polluted areas and low polluted areas based on the cut-off value, and used Fisher exact test to verify the cut-off value of air pollution. GAM was performed using ‘mgcv (Mixed GAM Computation Vehicle)’ package in R version 3.5.3, while other statistics were performed by the SPSS version 18.0. Significant results were considered at a *P* value lower than 0.05 (*P* < 0.05).

## Results

### Incidence of CH in China

**Fig. 1:**
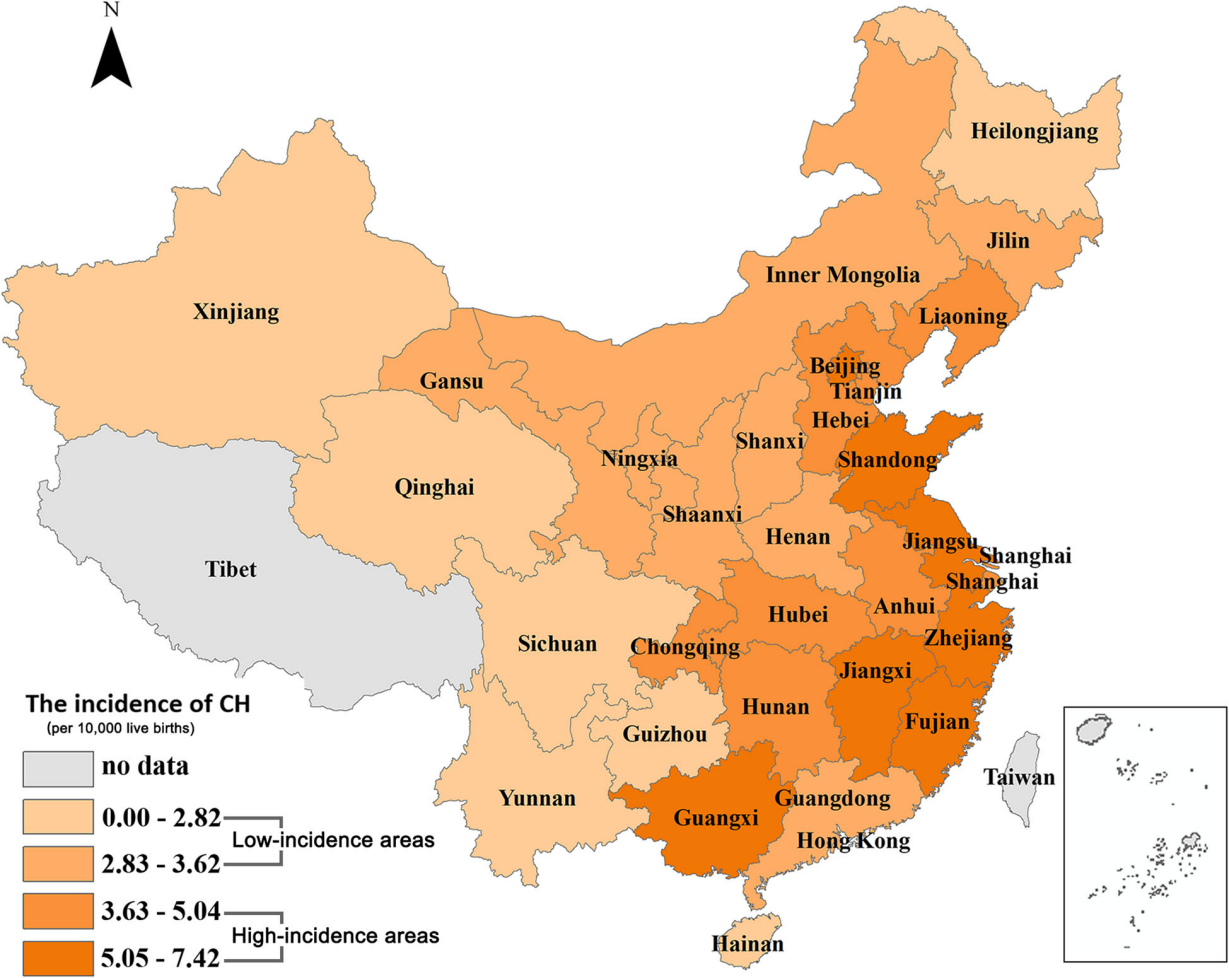
Average incidence of CH in 30 provinces of China from October 1, 2014 to October 1, 2015. ^a^ There was no data on Tibet, Taiwan, Hong Kong and Macau for no statistics. ^b^ Low-incidence areas meant that the incidence of CH was lower than 3.62 per 10,000 live births. ^c^ High-incidence areas meant that the incidence of CH was equal or greater than 3.62 per 10,000 live births

In 2015, approximately 15.1 million newborns were screened in China. A total of 6500 cases were identified as CH, which suggested an overall incidence rate of 4.31 per 10,000 screened newborns. The provinces with the highest incidence were mainly concentrated in the eastern coastal areas of China (Fig. 1), among which Zhejiang (7.42 per 10,000) and Fujian (7.34 per 10,000) were mainly affected regions. The incidence of CH in the central provinces was relatively lower, which was between 2.83 per 10,000 and 5.04 per 10,000. The provinces with the lowest incidence were mainly located in the Western areas, among which Xinjiang (1.88 per 10,000) had the lowest incidence of CH in China.

### Characteristics of exposure factors in China

Basic characteristic of CH and other pollutant indices were showed in Table 1**.** Moreover, it seemed that PM_2.5_ exposure and per capital GRP were weakly correlated with the incidence of CH, while spearman’s correlation coefficients were 0.350 and 0.434, separately. And there are strong correlations among various air pollutants (Table 2).

**Table 1:** Summary statistics of CH and other pollutant indices in China

Indicators ^a^	Mean	SD	P25	P50	P75
CH	4.049	4.570	2.818	3.615	5.038
PM_2.5_	58.131	16.842	48.142	57.458	67.688
PM_10_	100.611	27.491	81.083	102.292	116.938
AQI	88.958	20.746	77.023	89.015	103.312
Per capital GRP	51.458	22.089	35.113	41.221	63.904
Pb	2439.287	4218.210	129.150	784.650	3890.950
Hg	24.870	34.464	5.025	10.550	30.575
As	3484.770	7180.292	70.825	872.800	3387.625

*SD*: standard deviation; P25, P50, P75: 25th, 50th, 75th percentile

^a^ The unit of measurement for each indicator: per 10,000 for CH; μg/m^3^ for PM_2.5_ and PM_10_; thousand yuan for per capital GRP; million tons for Pb, Hg and As

**Table 2:** Spearman’s rank correlation analysis among various indicators in China

Indicators	CH	PM_2.5_	PM_10_	AQI	PCGRP	Pb	Hg	As
CH	1.000	0.350^a^	0.047	0.201	0.434*	0.156	−0.084	0.058
PM_2.5_		1.000	0.724*	0.904*	0.240	−0.096	− 0.196	− 0.168
PM_10_			1.000	0.931*	0.111	−0.049	0.054	−0.035
AQI				1.000	0.186	−0.074	− 0.056	−0.099
PCGRP					1.000	−0.291	−0.381*	− 0.342
Pb						1.000	0.803*	0.924*
Hg							1.000	0.805*
As								1.000

*PCGRP* Per capital gross regional product

* *p*<0.05

^a^ *p* = 0.058

### Associations between air pollution exposure and the incidence of CH

It was observed that an increase of 1 μg/m^3^ in average concentration of PM_2.5_ could increase the risk of CH (adjusted OR = 1.016 per 1 μg/m^3^ change, 95%CI, 1.001–1.031). However, significant associations were not found with regard to PM_10_ (adjusted OR = 1.009, 95%CI, 0.996–1.018) or AQI(adjusted OR = 1.012, 95%CI,0.998–1.026) and the risk of CH in the offspring (Table 3).

**Table 3:** Adjusted ORs^a^ (95% CIs) of PM_2.5_, PM_10_, AQI exposure and the incidence of CH

Air pollutants	*β*	*S.E*	OR (95%CI)
PM_2.5_	0.015	0.008	1.016 (1.001–1.031)*
PM_10_	0.007	0.006	1.009 (0.996–1.018)
AQI	0.012	0.007	1.012 (0.998–1.026)

^a^ Models were adjusted for per capital GRP and the content of heavy metal in wastewater

* *p*<0.05

### Approximate cut-off value of PM_2.5_ for decreasing the incidence of CH effectively

**Fig. 2:**
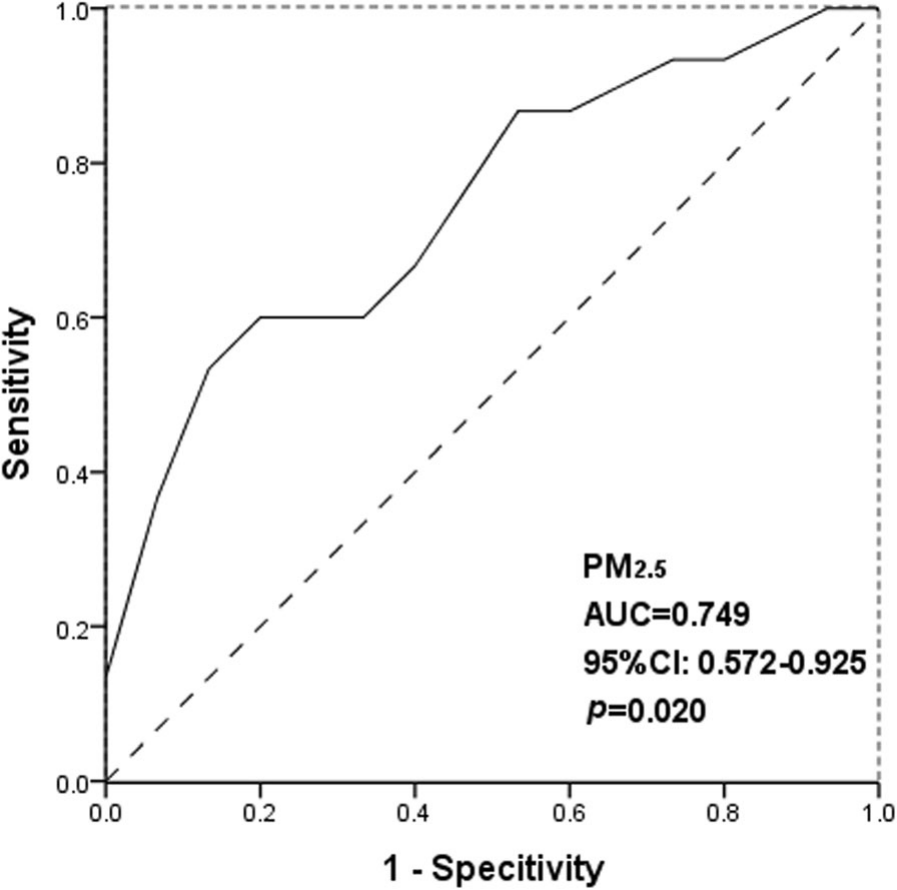
ROC curve of PM_2.5_ for predicting the risk CH. ^a^ AUC: Area under the curve, meant the area between the curve and the reference line

**Fig. 3:**
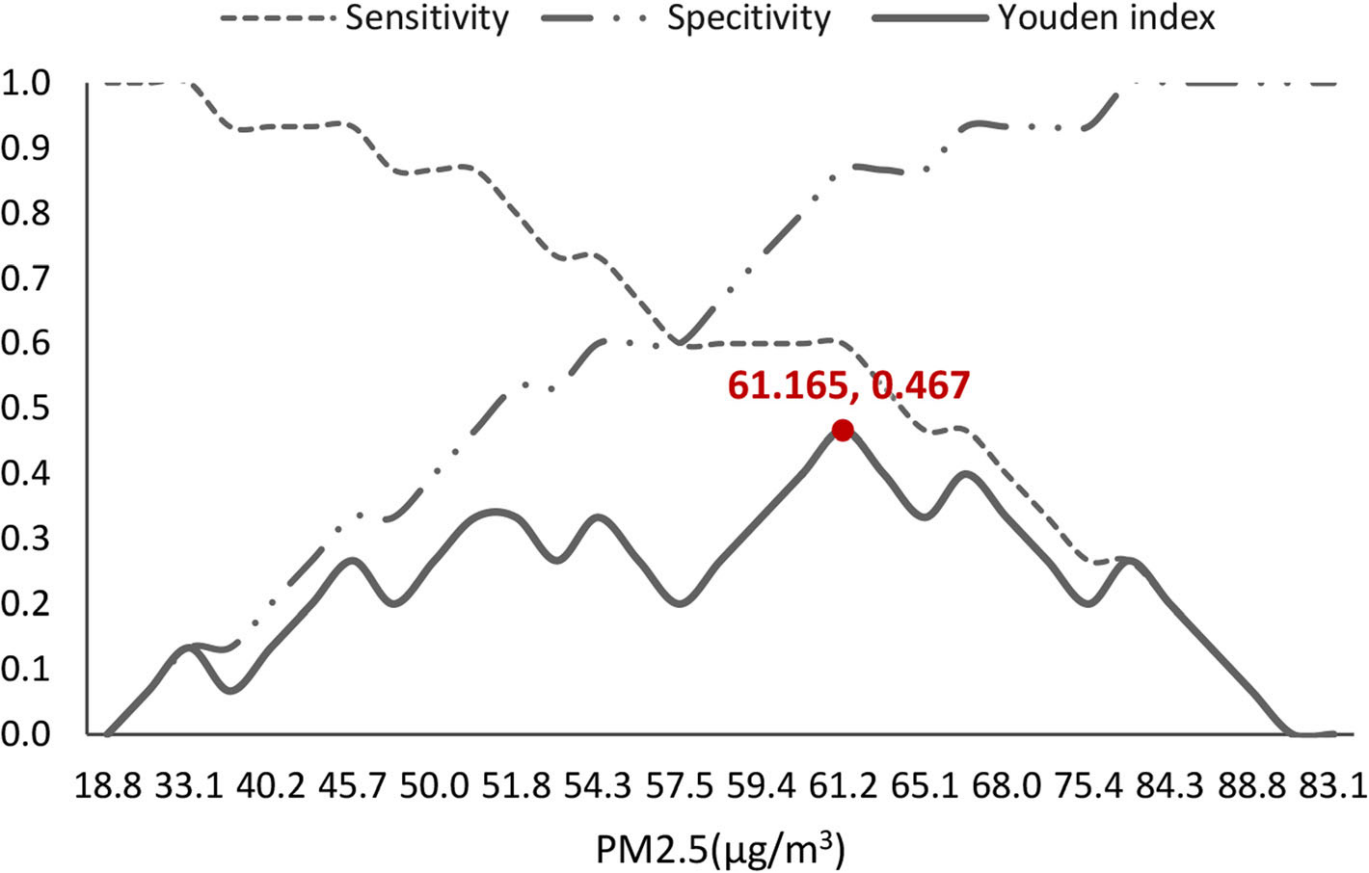
Sensitivity, specificity and Jordan index changes at different PM_2.5_ exposure concentrations

Areas with high-incidence had slightly higher levels of PM_2.5_ exposure (t = 2.407, *p* = 0.023) and per capital GRP (t = 2.805, *p* = 0.011) compared with those with low-incidence (Additional file 1: Table S1). Based on the positive relationship between prenatal PM_2.5_ exposure and the incidence of CH for the offspring, ROC curve was performed to identify cut-off value of prenatal exposure concentrations of PM_2.5_ for predicting and decreasing the risk of CH. Prenatal PM_2.5_ exposure was associated with the risk of CH with Area Under Curve (AUC) of 0.749 (95%CI, 0.572–0.926) (Fig. 2). By calculating Yoden index, the cutoff value of PM_2.5_ for predicting CH was 61.165 μg/m^3^, with a sensitivity of 0.600 and a specificity of 0.867(Fig. 3).

### Sensitivity analysis

In order to evaluate the stability of the above results, 30 provinces were divided into low-pollution areas (*n* = 18) and high-pollution areas (*n* = 12) based on the cut-off value of PM_2.5_ (61.165 μg/m^3^). Finally 12 out of 15 low-incidence areas were low-polluted, while 10 out of 15 high-incidence areas were high-polluted. The results of Fisher’s accuracy test showed that the degree of air pollution had a significant effect on the incidence of CH (*p* = 0.025) (Additional file 1: Table S2).

## Discussion

### Associations between PM_2.5_, PM_10_, AQI and CH risk in the offspring

The present study provided evidence that the high long-term PM_2.5_ exposure levels during pregnancy might be associated significantly with the elevated incidence of CH in the offspring. This association was not found with regard to PM_10_ and AQI. We further demonstrated an approximate cut-off value of PM_2.5_ (61.165 μg/m^3^) below which the risk of CH in the offspring could be effectively reduced.

### Maternal high exposure to PM_2.5_ might increase the risk of CH in the offspring

The present findings reported that per 1 μm/m^3^ particle increase in PM_2.5_ exposure during gestation was associated with the risk of CH in the offspring. The adjusted OR for this comparison was 1.016 (95% CI, 1.001–1.031). To the best of our knowledge, this was the first study to report on associations between maternal exposure to air pollution and the risk of CH in the offspring. The study by Janssen and colleagues conducted in Belgium suggested that PM_2.5_ exposure during the third trimester of gestation was inversely associated with TSH levels and the levels of the FT4/FT3 ratio in cord blood [37]. However, this study did not analyze the direct effect of PM_2.5_ exposure on the risk of CH in the offspring. In addition, a study that was conducted in animals demonstrated that outdoor as well as indoor airborne particulate matter (APM) extracts were able to strongly interfere with T4 binding to transthyretin (TTR) [38]. Thus, it was speculated that airborne particulate matter might have an effect on the newborn thyroid function. With the exception of these two reports, no other study has been conducted to examine the association between maternal exposure to air pollution and thyroid function in the offspring.

### PM_2.5_ may affect fetal thyroid function by interfering with placental nutrition

Previous studies reported that PM_2.5_ exposure might contribute to systemic oxidative stress, which might in turn lead to DNA damage and an increase in the number of placental DNA adducts [39–41]. Alternatively, PM_2.5_ might also bind to receptors required for placental growth factors leading to decreased fetal-placental exchange of oxygen and nutrients [42]. It may impact the growth of fetal endocrine system and thyroid function. In addition, it was found that PM exposure was associated with systemic inflammation and acute placental inflammation [43–46], whereas inflammation could be associated with inadequate placental perfusion. This reduced perfusion of the placenta can mediate placental inflammatory responses and its biologic sequelae, resulting in impaired transplacental nutrient exchange. Thus, we speculated that inadequate placental perfusion caused by PM2.5 exposure might cause fetal organ and tissue growth restriction in utero might contribute to interference with specific processes, such as the nutrition of the fetus and reduced oxygenation of maternal blood.

### Identification of an optimal cut-off value of PM_2.5_ for controlling CH

Through ROC curves, the cutoff value of PM_2.5_ for predicting CH for the offspring was calculated as 61.165 μg/m^3^, with a sensitivity of 0.600 and a specificity of 0.867. In previous studies, the dose-response relationship between air pollution and diseases was mainly explored on the respiratory system. A population-based study in China suggested that the cut-off value of PM_2.5_ was 83.0 μg/m^3^ for the prediction of acute exacerbation of chronic obstructive pulmonary disease (AECOPD) by ROC curves, which was higher than the corresponding value for PM_2.5_ noted in the present study (61.165 μg/m^3^) [47]. So it seemed that the interference of air pollution on maternal and fetal health may be more sensitive. Based on this, the effect of air pollution on maternal and fetal health should be taken into serious consideration. The results of the statistical analysis of the present study indicated that the monthly average concentration of PM_2.5_ in various provinces of China ranged from19.83 μg/m^3^ to 91.83 μg/m^3^, which was considerably higher than the environmental air quality standards recommended by the WHO (10 μg/m^3^) [3]. However, China lacks the guidelines to suggest an interim target for the gradual reduction of air pollution. Therefore, 61.165 μg/m^3^ for PM_2.5_ could be used as an interim target with regard to maternal and child health, which may be more suitable for high polluted areas that could aim to gradually reduce air pollution levels.

### Advantages and limitations

A main advantage of the present study was the combination of two databases to explore the association between air pollution exposure during gestation and the initial risk of CH in the offspring. Data analysis indicated that the incidence of CH was always higher in high PM_2.5_-exposed areas. However, a limitation of the present study should also be considered. We were unable to obtain the general demographic characteristics of the pregnant women directly from the Newborn Screening Centre. Thus, the present study did not adjust the potential risk factors for CH, such as gestational age, family history of thyroid function, maternal thyroid diseases, and iodine intake status, which may cause a certain bias in the results obtained. But per capital GRP and the content of heavy metals including Pb, Hg and As in wastewater in 30 provinces were adjusted in regression models in order to minimize bias as far as possible. In addition, although the cut-off value was not based on the global incidence of CH due to lack of authoritative data, it may provide a reference for other countries or areas with high incidence of CH [48, 49].

### Future perspectives

The present findings were indicative of high long-term exposure to PM_2.5_ levels that were associated significantly with an elevated incidence of CH in the offspring investigated.

Although the present study did not reveal an association between PM_10_ and AQI exposure levels and the risk of CH in the offspring, this is a possible association that could be examined in future studies. Certain reports have shown that PM_10_ also exhibits an adverse effect on the body and that it may increase the risk of adverse perinatal outcomes. Therefore, it is necessary to conduct epidemiological investigations based on specific human populations in order to analyze the key exposure window and the dose-response relationship between air pollution (PM_2.5_, PM_10_, NO_2_, CO, O_3_ and SO_2_) and thyroid function in the offspring. Furthermore, subsequent studies could examine and pinpoint the associations among air pollution, neonatal thyroid function and neurodevelopment in infants with particular emphasis on the molecular mechanism of thyroid metabolism.

## Conclusions

The present study suggested that PM_2.5_ exposure may exert a positive association with an increased risk of CH, and that the average concentration of PM_2.5_ during pregnancy should be less than 58.500 μg/m^3^. Our preliminary findings indicated that maternal exposure to air pollution might affect fetal thyroid development. The data can further aid the environmental administrative departments to formulate relevant policies in order to gradually improve air quality in highly polluted areas. Furthermore, the effect of air pollution on neonatal thyroid function and neurodevelopment should be explored and investigated in detail in further studies.

## Supplementary information

**Additional file 1: Table S1.** Comparison of exposure characteristics in low-incidence areas and high-incidence areas. **Table S2.** Fisher exact test of air pollution and the incidence of CH.

## Data Availability

The datasets generated during the current study are available in two open access website, including Chinese Maternal and Child Health Surveillance Network [http://www.mchscn.org/] and Chinese Air Quality Online Monitoring and Analysis Platform [https://www.aqistudy.cn/]. Additional data used during the current study are available from the corresponding author upon reasonable request.

## Abbreviations

AECOPD: Acute exacerbation of chronic obstructive pulmonary disease
APM: Airborne particulate matter
AQI: Air quality index
As: Arsenic
AUC: Area under curve
CH: Congenital hypothyroidism
CI: Confidence internal
FT4: Free thyroxine
GRP: Gross regional product
Hg: Mercury
OR: Odd ratio
PAHs: Polycyclic aromatic hydrocarbons
Pb: Lead
PM: Particulate matter
PM_10_: Inhalable particles
PM_2.5_: Fine particulate matter
ROC: Receiver operating characteristic
SD: Standard deviation
TSH: Thyrotropin
TTR: Transthyretin
WHO: World Health Organization

## References

[1] Shaddick G, Thomas ML, Amini H, Broday D, Cohen A, Frostad J, Green A, Gumy S, Liu Y, Martin RV (2018). Data integration for the assessment of population exposure to ambient air pollution for global burden of disease assessment. Environ Sci Technol.

[2] Tian Y, Liu H, Zhao Z, Xiang X (2018). Association between ambient air pollution and daily hospital admissions for ischemic stroke: a nationwide time-series analysis. PLoS Med.

[3] Organization WHO: Air quality guidelines: Global update (2005). Particulate matter, ozone, nitrogen dioxide and sulfur dioxide. Indian J Med Res.

[4] China MoEEotPsRo.. In: Chinese ecological and environment bulletin. [cited 17 July 2018]Available from: http://www.mee.gov.cn/2017.

[5] Lin H, Wang X, Liu T, Li X, Xiao J, Zeng W, Ma W (2017). Air pollution and mortality in China. Adv Exp Med Biol.

[6] Day DB, Xiang J, Mo J, Li F, Chung M, Gong J, Weschler CJ, Ohman-Strickland PA, Sundell J, Weng W (2017). Association of Ozone Exposure with Cardiorespiratory Pathophysiologic Mechanisms in healthy adults. JAMA Intern Med.

[7] Kaufman JD, Adar SD, Barr RG, Budoff M, Burke GL, Curl CL, Daviglus ML, Diez Roux AV, Gassett AJ, Jacobs DR (2016). Association between air pollution and coronary artery calcification within six metropolitan areas in the USA (the Multi-Ethnic Study of Atherosclerosis and Air Pollution): a longitudinal cohort study. Lancet.

[8] Hoek G, Krishnan RM, Beelen R, Peters A, Ostro B, Brunekreef B, Kaufman JD (2013). Long-term air pollution exposure and cardio- respiratory mortality: a review. Environ Health.

[9] Cohen AJ, Brauer M, Burnett R, Anderson HR, Frostad J, Estep K, Balakrishnan K, Brunekreef B, Dandona L, Dandona R (2015). Estimates and 25-year trends of the global burden of disease attributable to ambient air pollution: an analysis of data from the global burden of diseases study. Lancet.

[10] Vidale S, Campana C (2018). Ambient air pollution and cardiovascular diseases: from bench to bedside. Eur J Prev Cardiol.

[11] Landrigan PJ (2017). Air pollution and health. Lancet Public Health.

[12] Ranathunga N, Perera P, Nandasena S, Sathiakumar N, Kasturiratne A, Wickremasinghe R (2019). Effect of household air pollution due to solid fuel combustion on childhood respiratory diseases in a semi urban population in Sri Lanka. BMC Pediatr.

[13] Lelieveld J, Evans JS, Fnais M, Giannadaki D, Pozzer A (2015). The contribution of outdoor air pollution sources to premature mortality on a global scale. Nature.

[14] Dauchet L, Hulo S, Cherot-Kornobis N, Matran R, Amouyel P, Edme JL, Giovannelli J (2018). Short-term exposure to air pollution: associations with lung function and inflammatory markers in non-smoking, healthy adults. Environ Int.

[15] Hegewald MJ, Crapo RO (2011). Respiratory physiology in pregnancy. Clin Chest Med.

[16] Wang Q, Liu M, Yu Y, Li Y (2016). Characterization and source apportionment of PM2.5-bound polycyclic aromatic hydrocarbons from Shanghai city, China. Environ Pollut.

[17] Li Y, Zhang Z, Liu H, Zhou H, Fan Z, Lin M, Wu D, Xia B (2016). Characteristics, sources and health risk assessment of toxic heavy metals in PM2.5 at a megacity of Southwest China. Environ Geochem Health.

[18] Sun X, Luo X, Zhao C, Zhang B, Tao J, Yang Z, Ma W, Liu T (2016). The associations between birth weight and exposure to fine particulate matter (PM2.5) and its chemical constituents during pregnancy: a meta-analysis. Environ Pollut.

[19] Dong X, Wang Q, Peng J, Wu M, Pan B, Xing B (2018). Transfer of polycyclic aromatic hydrocarbons from mother to fetus in relation to pregnancy complications. Sci Total Environ.

[20] Ollivon D, Blanchoud H, Motelay-Massei A, Garban B (2002). Atmospheric deposition of PAHs to an urban site, Paris. France Atmospheric Environment.

[21] Hansen CA, Barnett AG, Jalaludin BB, Morgan GG (2009). Ambient air pollution and birth defects in Brisbane, Australia. PLoS One.

[22] Tanner JP, Salemi JL, Stuart AL, Yu H, Jordan MM, DuClos C, Cavicchia P, Correia JA, Watkins SM, Kirby RS (2015). Associations between exposure to ambient benzene and PM(2.5) during pregnancy and the risk of selected birth defects in offspring. Environ Res.

[23] Habermann M, Gouveia N (2014). Socioeconomic position and low birth weight among mothers exposed to traffic-related air pollution. PLoS One.

[24] Rashid AA, Devaraj NK (2018). Oh no! Now I have diabetes. Rawal Med J.

[25] Devaraj NK. A recurrent cutaneous eruption. BMJ Case Rep. 2019:12(2):e228355. 10.1136/bcr-2018-228355 PMC6366803 30709894

[26] Anand MR, Ramesh P, Nath D (2015). Congenital hypothyroidism screening with umbilical cord blood: retrospective analysis. Indian Pediatr.

[27] Yi Mou JZ. [cited 1 March 2018]. In: National Maternal and Child Health Monitoring and Annual Report Available from: http://www.mchscnorg/, 2017.

[28] Fan X, Chen S, Qian J, Sooranna S, Luo J, Li C, Tang Q, Lin C (2015). Incidence and Interrelated Factors in Patients With Congenital Hypothyroidism as Detected by Newborn Screening in Guangxi, China. Glob Pediatr Health.

[29] Lof C, Patyra K, Kuulasmaa T, Vangipurapu J, Undeutsch H, Jaeschke H, Pajunen T, Kero A, Krude H, Biebermann H (2016). Detection of novel gene variants associated with congenital hypothyroidism in a Finnish patient cohort. Thyroid.

[30] Singh A, Purani C, Mandal A, Mehariya KM, Das RR (2016). Prevalence of thyroid disorders in children at a tertiary Care Hospital in Western India. J Clin Diagn Res.

[31] Li M, Eastman CJ (2010). Neonatal TSH screening: is it a sensitive and reliable tool for monitoring iodine status in populations?. Best Pract Res Clin Endocrinol Metab.

[32] Khammarnia M, Siakhulak FR, Ansari H, Peyvand M (2018). Risk factors associated with congenital hypothyroidism: a case-control study in Southeast Iran. Electron Physician.

[33] Chinese Air Quality Online Monitoring. https://www.aqistudy.cn/. Accessed 20 May 2018.

[34] Ministry of Ecology and Environment of the People’s Republic of China. http://www.mee.gov.cn/. Accessed 12 May 2018.

[35] China Statistical Yearbook[M]. Chinese national bureau of statistics. 2015. http://www.stats.gov.cn/tjsj/ndsj/2015/indexch.htm

[36] China Statistical Yearbook[M]. Chinese national bureau of statistics. 2016. http://www.stats.gov.cn/tjsj/ndsj/2016/indexch.htm

[37] Janssen BG, Saenen ND, Roels HA, Madhloum N, Gyselaers W, Lefebvre W, Penders J, Vanpoucke C, Vrijens K, Nawrot TS (2017). Fetal thyroid function, birth weight, and in utero exposure to fine particle air pollution: a birth cohort study. Environ Health Perspect.

[38] Alink GM, Brouwer A, Heussen GA (1994). Effects of outdoor and indoor airborne particulate matter on thyroid hormone and vitamin a metabolism. Toxicol Lett.

[39] Damasceno DC, Netto AO, Iessi IL, Gallego FQ, Corvino SB, Dallaqua B, Sinzato YK, Bueno A, Calderon IM, Rudge MV (2014). Streptozotocin-induced diabetes models: pathophysiological mechanisms and fetal outcomes. Biomed Res Int.

[40] Zhou J, Luo J, Zhao H, Wang J, Lin F, Zhang H, Su Y, Chen Y, Zeng Y, Lin Q (2015). Risk factors of 125 cases of neonatal congenital hypothyroidism during perinatal period. Zhonghua Liu Xing Bing Xue Za Zhi.

[41] Huang HB, Lai CH, Chen GW, Lin YY, Jaakkola JJ, Liou SH, Wang SL (2012). Traffic-related air pollution and DNA damage: a longitudinal study in Taiwanese traffic conductors. PLoS One.

[42] van den Hooven EH, Pierik FH, de Kluizenaar Y, Hofman A, van Ratingen SW, Zandveld PY, Russcher H, Lindemans J, Miedema HM, Steegers EA (2012). Air pollution exposure and markers of placental growth and function: the generation R study. Environ Health Perspect.

[43] Lee PC, Talbott EO, Roberts JM, Catov JM, Sharma RK, Ritz B (2011). Particulate air pollution exposure and C-reactive protein during early pregnancy. Epidemiology.

[44] Panagiotakos DB, Pitsavos C, Chrysohoou C, Skoumas J, Masoura C, Toutouzas P (2004). Stefanadis C, study a: effect of exposure to secondhand smoke on markers of inflammation: the ATTICA study. Am J Med.

[45] Lane KJ, Levy JI, Scammell MK, Peters JL, Patton AP, Reisner E, Lowe L, Zamore W, Durant JL, Brugge D (2016). Association of modeled long-term personal exposure to ultrafine particles with inflammatory and coagulation biomarkers. Environ Int.

[46] Vadillo-Ortega F, Osornio-Vargas A, Buxton MA, Sanchez BN, Rojas-Bracho L, Viveros-Alcaraz M, Castillo-Castrejon M, Beltran-Montoya J, Brown DG, O'Neill MS (2014). Air pollution, inflammation and preterm birth: a potential mechanistic link. Med Hypotheses.

[47] Sun XW, Chen PL, Ren L, Lin YN, Zhou JP, Ni L, Li QY (2018). The cumulative effect of air pollutants on the acute exacerbation of COPD in Shanghai, China. Sci Total Environ.

[48] Anastasovska V, Kocova M (2017). Ethnicity and incidence of congenital hypothyroidism in the capital of Macedonia. J Pediatr Endocrinol Metab.

[49] Mitrovic K, Vukovic R, Milenkovic T, Todorovic S, Radivojcevic J, Zdravkovic D (2016). Changes in the incidence and etiology of congenital hypothyroidism detected during 30 years of a screening program in Central Serbia. Eur J Pediatr.

