# Supplementary material for: Maternal exposure to PM2.5 may increase the risk of congenital hypothyroidism in the offspring: a national database based study in China
Source: BMC Public Health. 2019 Nov 19;19:1412. doi: 10.1186/s12889-019-7790-1 (PMC6862828; doi:10.1186/s12889-019-7790-1)
Supplement: Supplementary file 1 — Additional file 1: Table S1. Comparison of exposure characteristics in low-incidence areas and high-incidence areas. Table S2. Fisher exact test of air pollution and the incidence of CH. [file 12889_2019_7790_MOESM1_ESM.docx]

**Additional file 1**

**Table S1. Comparison of exposure characteristics in low-incidence areas and high-incidence areas**

| Indicators | Low-incidence areas  (Mean ± SD) | High-incidence areas  (Mean ± SD) | *t* | *p* |
| --- | --- | --- | --- | --- |
| PM_2.5_ | 51.182±14.563 | 64.878±16.539 | 2.407 | 0.023 |
| PM_10_ | 98.522±29.075 | 102.700±26.662 | 0.410 | 0.685 |
| AQI | 83.467±19.685 | 94.450±20.963 | 1.479 | 0.150 |
| per capital GRP | 41.285±12.631 | 61.631±25.088 | 2.805 | 0.011 |
| Pb | 17.395±21.781 | 31.391±55.735 | 0.906 | 0.373 |
| Hg | 0.220±0.226 | 0.278±0.439 | 0.457 | 0.651 |
| As | 23.176±41.264 | 46.513±93.194 | 0.887 | 0.383 |

^a^ The unit of measurement for each indicator: μg/m^3^ for PM_2.5_ and PM_10_; thousand yuan for per capital GRP; million tons for Pb, Hg and As.

SD: standard deviation.

* *p*＜0.05

# Table S2. Fisher exact test of air pollution and the incidence of CH

| Pollution | Low-incidence areas | High-incidence areas | Total | *p* |
| --- | --- | --- | --- | --- |
| Low-polluted areas | 12 | 5 | 17 |  |
| High-polluted areas | 3 | 10 | 13 |  |
| Total | 15 | 15 | 30 | 0.025 |

Low-incidence areas meant that the incidence of CH was lower than 3.62 per 10,000 live births; high-incidence areas meant that the incidence of CH was equal or greater than 3.62 per 10,000 live births. Low-polluted areas meant that the exposure concentration of PM_2.5_ was lower than 61.165μg/m^3^; high-polluted areas meant that the exposure concentration of PM_2.5_ was equal or greater than 61.165μg/m^3^.
